# Environmental, Spatial, and Sociodemographic Factors Associated with Nonfatal Injuries in Indonesia

**DOI:** 10.1155/2017/5612378

**Published:** 2017-04-03

**Authors:** Sri Irianti, Puguh Prasetyoputra

**Affiliations:** ^1^National Institute of Health Research and Development, Ministry of Health, Jl. Percetakan Negara No. 29, Jakarta Pusat 10560, Indonesia; ^2^Research Center for Population, Indonesian Institute of Sciences (P2K-LIPI), Jl. Jend. Gatot Subroto No. 10, Jakarta Selatan 12710, Indonesia

## Abstract

*Background*. The determinants of injuries and their reoccurrence in Indonesia are not well understood, despite their importance in the prevention of injuries. Therefore, this study seeks to investigate the environmental, spatial, and sociodemographic factors associated with the reoccurrence of injuries among Indonesian people.* Methods*. Data from the 2013 round of the Indonesia Baseline Health Research (IBHR 2013) were analysed using a two-part hurdle regression model. A logit regression model was chosen for the* zero-hurdle part*, while a zero-truncated negative binomial regression model was selected for the* counts part*. Odds ratio (OR) and incidence rate ratio (IRR) were the measures of association, respectively.* Results*. The results suggest that living in a household with distant drinking water source, residing in slum areas, residing in Eastern Indonesia, having low educational attainment, being men, and being poorer are positively related to the likelihood of experiencing injury. Moreover, being a farmer or fishermen, having low educational attainment, and being men are positively associated with the frequency of injuries.* Conclusion*. This study would be useful to prioritise injury prevention programs in Indonesia based on the environmental, spatial, and sociodemographic characteristics.

## 1. Introduction

Injuries and violence are a public health threat worldwide. They are attributable to 9% of global deaths, equivalent to more than five million annual mortality; more than 90% of these injury-related deaths occur in low- and middle-income countries (LMICs) [1]. Despite these figures, injuries are still neglected in developing countries [2, 3].

Studies from around the world have shown a negative relationship between socioeconomic status (SES) and childhood injury morbidity and mortality [4]. Moreover, Kim and colleagues' [5] and Yiengprugsawan and colleagues' [6] studies found a negative relationship between income and injury experience. Furthermore, poor housing conditions are associated with a broad range of health conditions, including injuries and mental health [7].

Besides health implications, injuries can also have economic repercussions. Keall and colleagues [8] found that the annual social cost of unintentional home injuries was more than threefold that of the annual social cost of road injury. Moreover, workers who return after experiencing injuries may have lower wages [9]. Furthermore, injury experience may degrade cognitive ability which may later affect the capacity to work [10].

According to the report of the latest Indonesia Baseline Health Research (IBHR), the prevalence of injury in Indonesia increased from 7.5% to 8.2% [11]. Despite this worrying increase, research on injuries in Indonesia is limited. In 2009, using data from the 2007 round of the IBHR, Riyadina and coworkers [12] investigated the sociodemographic determinants of road traffic injury (RTI) in Indonesia. They reported a significant relationship between age, sex, education, employment status, living in urban area, and wealth quintiles with the experience of RTI. Another more recent study by Tana and Ghani [13] analysed the 2013 wave of the IBHR to examine the determinants of injury among productive-age workers in Indonesia. They found that teenagers and males were more prone to experiencing injuries. However, these studies were of similar limitation: they used binary logistic regression to model the determinants and thus missed the information on the frequencies of injuries collected in the IBHR.

Improving the capacity of the Government of Indonesia in preventing injury is paramount [14], and reliable research related to the drivers of injuries is a significant input. However, studies from low- and middle-income countries are limited [15]. Therefore, this study seeks to investigate the factors associated with the reoccurrence of injuries among Indonesians. Specifically, this study explores the relationship between environmental, spatial, and sociodemographic characteristics and injury experience. The rest of the article is organized as follows. The next section describes the data source and econometric method used. The section afterward presents the main empirical findings. The penultimate section discusses those findings. The last part then concludes.

## 2. Material and Methods

### 2.1. Data Source

The data were drawn from the IBHR 2013, a survey managed by the National Institute of Health Research and Development (NIHRD) of the Ministry of Health of the Republic of Indonesia. The 2013 wave includes 1,027,763 individuals from 294,959 households and is representative of the 33 provinces at the time of the survey [11]. The NIHRD has obtained informed consent from the respondents before interviews and preserved their anonymity. Further details on ethical and sampling procedures can be read elsewhere [11].

### 2.2. Outcome Measures

The World Health Organization defines injury as the physical damage that emanates when a human body is exposed to intolerable levels of energy in a sudden or brief manner and can be categorised into three types: (1) unintentional, (2) intentional, and (3) undetermined intent [16]. The IBHR follows that definition. However, it only collected data on “unintentional” injuries. Moreover, the further questions on injuries refer to the injury that was deemed the most severe by the respondent.

In the 2013 round of IBHR, there are two main questions on injury. The first was “in the past 12 months, have you had any events (accidents, violence, and falls) that were severe enough to interfere with daily activities?” This question seeks to find the annual injury prevalence (a form of period prevalence). This question was also the source of the first dependent variable representing injury prevalence where a “Yes” response is coded as “1” and a “No” response is coded as “0.” Then the second question was “if yes, in the past 12 months, how many times you were injured?” The second dependent variable, injury occurrence, is taken from the second question, which was the number of injuries experienced by the respondents during the last 12 months preceding the survey. This question seeks to measure the annual injury incidence (a form of cumulative incidence). The reported number of injuries was not restricted to certain types of injuries. Hence, as long as the injury interferes with the daily activity, it will be counted.

### 2.3. Explanatory Variables

The selection of explanatory variables in this study follows Peek-Asa and Hyder's [14] framework for the epidemiological study of traumatic injury. As in any other epidemiological model, there exists an agent-host interaction. In the case of injuries, the agent is energy. A host potentially can be injured when exposed to this energy, which comes in many forms like mechanical, electrical, chemical, radiation, and thermal [14]. Moreover, the explanatory variables chosen were also selected based on previous observational studies [5, 6, 17] and were classified into three groups, namely, environmental, spatial, and sociodemographic characteristics. There are two environmental variables. The first one, distance to drinking water source, was classified into water source on premise, ≤100 metres away, 101–1000 metres, or >1000 metres. The second one, slum residence, was either household residing in a slum area or not. This information was obtained from the observation of the enumerators. There are two spatial indicators in this study: region of residence (Java-Bali, Sumatera, or Eastern Indonesia) and place of residence (urban or rural area).

There are five sociodemographic variables in this study. Sex was classified as female or male. Age was in 10-year groups: 10–19, 20–29, 30–39, 40–49, 50–59, and ≥60 years. Marital status was categorised into five groups: never married (≥15 years), never married (<15 years), currently married/living together, bereaved, or divorced/separated. Occupation was categorised into five groups: students, unemployed, employed, farmer/fishers, or entrepreneur. Education was categorised into six groups: none, some primary, primary, junior high, senior high, and college or university. Lastly, there are two economic variables. The first one is the* Raskin* variable, which was classified as has not ever received/bought or has ever received/bought. Raskin or short for* “beras untuk rumah tangga miskin”* (rice for poor households) is a national program designed to make rice more affordable for the poor to increase food security [18].

Moreover, the second economic variable is household wealth represented by wealth index scores. The IBHR 2013 already provided a wealth index, which has been converted into wealth quintiles, as a proxy for household affluence [11]. However, as the sample has been restricted to individuals aged 10 and above, it is imperative that a new wealth index is calculated; using polychoric principal component analysis (PCA) to obtain scores from relevant variables [19, 20]. The scores were then used to weight the variables to get the scores for the indices. The variables included in the polychoric PCA analysis were the main material of floor, the main material of wall, type of cooking fuel, and ownership of household assets (bicycle, motorcycle, car, cable TV, air conditioner, water heater, 12 kg or higher gas cylinder, and refrigerator).

### 2.4. Data Analysis

In this study, the data analysis comprises two parts, descriptive analysis and multivariable analysis. Individuals younger than ten years old were excluded from the analysis as questions related to main past activities (not working, working, looking for a job, or studying) were only administered to those individuals in that age range; also excluded were those with missing information on the independent variables (14,647 individuals). These exclusions yield a complete-case final analytic sample of 822,709 individuals.

The abundance of zeroes in the data leads to overdispersion (i.e., the variance significantly exceeds the mean) [21]. This phenomenon is further confirmed by fitting a Poisson regression model and examining the Lagrange multiplier (LM) statistic (results not shown for the sake of brevity) [22]. This characteristic prohibits the use of models that rely on the assumption that the data follows a Poisson distribution. One of the econometric models that can control overdispersion is the hurdle model [22, 23]. Cragg [24] initially thought of this model, and, later, its application was introduced by Mullahy [25].

The basic principle of a hurdle model is to separate the model into two parts. The first part explains the generation of positive counts (coded as 1) as opposed to zero counts (coded as 0), and the second part explains the generation of the nonzero counts [22]. In this study, the first part (i.e., the* zero-hurdle part*) is modelled using a binary logit regression. As for the nonzero counts section (i.e., the* counts part*), however, the choice of model is between the Poisson hurdle (PH) model and the negative binomial (NBH) model (for a detailed explanation, see Loeys and coworkers [26]).

Choosing the model for the* counts part* component was based on Akaike information criterion (AIC); the lower the value of AIC, the better the model [22]. The values of AIC statistics and AIC*n* (AIC statistic times the sample size) of the models compared can be seen in Table 1. Since the second model (the negative binomial logit hurdle nodel) has lower AIC statistics, it is chosen over the other. This model has been used in previous studies with topics besides injuries (see Bethell et al. [27]; Hellemans et al. [28]; Rose et al. [29]). A negative binomial logit hurdle model was then fitted to the data (using the “hnblogit” command; Hilbe [30]), with statistical significance evaluated at 1%, 5%, and 10% levels of significance.

In this study, the* zero-hurdle part* assesses the effect of an independent variable (e.g., household wealth) on the likelihood of experiencing at least one injury over the past 12 months. Moreover, the* counts part* assesses the effect of this independent variable on the frequency of injury experiences among those who are injured during the past 12 months. While the results of the* zero-hurdle part*, that is, the logit regression, are presented in odds ratios (ORs), the results of the* counts part*, that is, the zero-truncated negative binomial model, are reported in the form of incidence rate ratios (IRRs). All of the econometric analyses were conducted using Intercooled STATA version 13.1 [31].

## 3. Results and Discussion

### 3.1. Descriptive Statistics of the Sample

Simple descriptive statistics of the dependent and independent variables in the form of mean, standard deviation, minimum value, and maximum value were calculated and presented in Table 2. It can be seen that only 7.76% of individuals reported having been injured at least once during the last 12 months preceding the survey. This figure is considerably low as the IBHR only collected information on injuries that were severe enough to interfere with the daily activities of the respondent. Of those, the mean frequency of injuries is just below 1.46 times (not shown in Table 2). Moreover, the maximum number of injuries reported was 48 times in the past 12 months. Further analysis found that the respondent who reported having been injured four times in every month was a fisherman who is more prone to injuries due to the daily occupational hazard.

Approximately 61.40% of the individuals live in a household with drinking water source on premise, 31.18% of the individuals live in a household with drinking water source located less than 100 metres away, and the rest of the individuals live in a household with drinking water source located more than 100 metres away. Concerning slum residence, 15.90% of the individuals live in slum areas. Moreover, 34.44% of individuals reside in Java-Bali region, 32.97% live in Sumatera, and the rest (32.59%) live in Eastern Indonesia. Furthermore, less than half of the individuals (45.86%) reside in urban areas.

Regarding sex, just under half of the individuals are males (48.58%). Almost one in four of the individuals are of 10–19 years of age (23.72%), while the least proportion is in the over-60 age group with 10.58%. Concerning marital status, the individuals are predominantly married or living together constituting 60.12% of the sample. Regarding occupation, the majority of the individuals reported being unemployed (30.71%).

As for educational attainment, less than a third of individuals reported having completed primary education, while only 6.10% reported having attained a college or university qualification. More than half of the individuals live in a household which either has bought or received rice in the rice for the poor program (52.03%). Lastly, the mean of the wealth index score (not shown in Table 2) is close to zero (0.08) which is the common value of index generated using polychoric PCA [19].

### 3.2. Results of the Two-Part Model

Table 3 summarises the estimates of the two-part negative binomial logit hurdle model. The one on the left is the results of the* zero-hurdle part*, while the one the right is the results of the* counts part*. The overall model was highly significant (Wald *χ*^2^ = 11318.91;* P *< 0.001).

#### 3.2.1. Spatial Correlates

Both spatial variables, the region of residence and place of residence, were observed to be significantly associated with injury prevalence. Individuals living in Java-Bali region were at higher risk of experiencing injury (OR 1.39, 95% CI 1.36–1.42) compared to those residing in Sumatera. Likewise, people residing in Eastern Indonesia were more likely to suffer injury compared to the reference category (OR 1.56, 95% CI 1.53–1.59). Furthermore, living in Java-Bali region, as opposed to residing in Sumatera, was found to be significantly associated with higher incidence of injury (IRR 1.40, 95% CI 1.31–1.50). Likewise, living in Java-Bali region, as opposed to living in Sumatera, was found to be significantly associated with higher incidence of injury (IRR 1.13, 95% CI 1.06–1.21). These relationships are consistent with the study conducted by Tana and Ghani [13], which found the same relationship between the region of residence with an injury. Also, living in urban areas was significantly associated with the prevalence of injury (OR 1.03, 95% CI 1.01–1.05). This finding is consistent with the findings from Riyadina and coworkers [12] and Tana and Ghani [13]. Lastly, living in urban areas was associated with higher frequency of injury; however, this relationship was not significant (IRR 1.04, 95% CI 0.95–1.16).

#### 3.2.2. Environmental Correlates

Both of the environmental correlates were observed to be significantly related to annual injury prevalence and annual injury incidence. The further the drinking water source from home, the more likely the individuals to be injured (OR 1.14, 95% CI 1.10–1.18). However, this was not found to be significantly associated with the incidence of injury. Moreover, people living in slum areas were found to have a higher likelihood of being injured (OR 1.09, 95% CI 1,07–1.12) and have a higher incidence of injury (IRR 1.06, 95% CI 1.00–1.13). These results indicate that poor living conditions and lack of basic amenities may increase injury risk. Slum areas are also familiar with overcrowding which may spur the potential for specific injury types such as burn [32].

#### 3.2.3. Sociodemographic Correlates

All the six sociodemographic variables were observed to be significantly related to the probability of being injured; they are sex, age, marital status, occupation, education, and participation in Raskin program. Consistent with the extant literature [12, 13], males were found to be of higher odds of getting injured (OR 1.65, 95% CI 1.62–1.68) and a higher incidence of injury (IRR 1.14, 95% CI 1.07–1.20). Moreover, an increase in age was observed to be related to both injury risk and injury frequency where higher age corresponds to lower injury risk and injury frequency. This relationship is consistent with the extant literature [33–35] as younger people tend to be more careless than older ones.

In terms of marital status, never married (age ≥ 15 years, OR 1.33, 95% CI 1.29–1.37), bereaved (OR 1.30, 95% CI 1.24–1.36), and divorced individuals (OR 1.25, 95% CI 1.17–1.34) were found to be associated with higher odds of getting injured compared to married individuals. Furthermore, never married (age ≥ 15 years, IRR 1.12, 95% CI 1.02–1.24; age < 15 years, IRR 1.17, 95% CI 1.02–1.33) and divorced individuals (IRR 1.27, 95% CI 1.04–1.54) were found to be associated with higher incidence of injury compared to married individuals. This relationship is similar to what Tana and Ghani [13] found.

As for occupation of individuals, being employed, a student, a farmer, and an entrepreneur is significantly associated with higher likelihood of injury, as opposed to being unemployed. Kim and coworkers [5] found that agriculture workers have higher odds compared to unemployed (OR 1.06, 95% CI 1.03–1.09). However, being employed, a student, and an entrepreneur is significantly associated with lower injury incidence, as opposed to being unemployed. This finding is similar to what Tana and Ghani [13] found where the risk of injury also differs by type of occupation.

The last sociodemographic variable in this study is educational attainment. In line with previous research [5, 13], education was observed to be significantly associated with both annual injury prevalence and injury incidence. More specifically, the lower the educational attainment of an individual, the higher the odds of getting injured and the higher the injury incidence.

#### 3.2.4. Economic Correlates

The extant literature consistently has shown a negative relationship between economic status, in the form of household income or household wealth, and risk [5, 12, 13]. In this study, there are two economic variables. Participation in the Raskin program was significantly associated with the prevalence of injury (OR 1.12, 95% CI 1.10–1.14) but not with the incidence of injury (IRR 1.00, 95% CI 0.94–1.06). Moreover, wealth index was not significantly associated with the prevalence of injury (OR 1.01, 95% CI 0.99–1.01) but was significantly associated with the incidence of injury (IRR 0.96, 95% CI 0.94–0.98).

### 3.3. Study Limitations and Strengths

There are some limitations for this study. The variables used in this study were obtained from interviews using structured questionnaires. Hence, recall biases may arise when the respondents are asked to remember events which happened over an extended period (i.e., in the past 12 months). Moreover, owing to the nature of such lengthy retrospective assessment of outcomes, and the cross-sectional form of the IBHR 2013, there may be some problems with reverse causation between the independent variables and injury outcomes which limit the examination of causal relationships between these variables. Also, it is worth noting that several of the explanatory variables may not be exogenous. One example is the distance to drinking water source, which, in this study, assumed to be exogenous. This assumption is another limitation of and may lead to inefficiency of the regression model. Overall, one should keep these limitations in mind when interpreting the results of this study.

Despite bearing several previously mentioned drawbacks, this study also possesses a couple of advantages. First, the large data set used in this study provides substantial statistical power. Second, the statistical model employed in this study yields more information than the ones used in previous studies in the context of Indonesia.

## 4. Conclusion

This study attempts to address the spatial, environmental, and sociodemographic factors associated with the frequency of injuries among Indonesian people. Despite the limitations above, this study reveals that living in a household with distant drinking water source, residing in slum areas, residing in Eastern Indonesia, having low educational attainment, and being poorer are positively related to the likelihood of experiencing injury. This study also shows that job types correspond to a different risk of injury. Moreover, low educational attainment is positively associated with the frequency of injuries. Furthermore, the findings also emphasise that, compared to women, men are more vulnerable to experiencing repeated injuries over the year. This study would be useful to prioritise injury prevention programs based on the environmental, spatial, sociodemographic, and economic characteristics. Also, future studies that assess the determinants of injury in a longitudinal manner are recommended.

## Figures and Tables

**Table 1:** Akaike information criterion statistics of the hurdle models.

Number	Hurdle model	AIC*n*	AIC Statistic
1	Poisson logit hurdle (PH)	567160.75	0.6911461
2	Negative binomial logit hurdle (NBH)	545512.06	0.6647649

*Notes*. AIC, Akaike information criterion; AIC*n*, AIC statistic times the sample size.

Source is authors' calculation of the IBHR 2013 data.

**Table 2:** Descriptive statistics of the study variables (*N* = 820,609).

Variables	Number	Percent
Dependent variable		
Had at least one injury in the past 12 months		
No	756,942	92.24
Yes	63,667	7.76

Spatial		
Region of residence		
Sumatera	282,633	34.44
Java-Bali region	270,560	32.97
Eastern Indonesia	267,416	32.59

Place of residence		
Rural area	444,300	54.14
Urban area	376,309	45.86

Environmental		
Distance to drinking water source (DWS)		
DWS on premise	503,835	61.4
≤100 metres	255,884	31.18
101–1000 metres	52,586	6.41
>1000 metres	8,304	1.01

Household residing in a slum area		
No	690,162	84.1
Yes	130,447	15.9

Sociodemographic		
Sex		
Female	421,946	51.42
Male	398,663	48.58

Age (in years)		
10–19	194,675	23.72
20–29	126,306	15.39
30–39	156,091	19.02
40–49	149,526	18.22
50–59	107,169	13.06
≥60	86,842	10.58

Marital status		
Currently married/living together	493,315	60.12
Never married and age ≥ 15 years	163,876	19.97
Never married and age < 15 years	109,016	13.28
Bereaved	42,040	5.12
Divorced/separated	12,362	1.51

Occupation		
Unemployed (Ref.)	251,992	30.71
Student	141,008	17.18
Employed	165,686	20.19
Farmer	159,928	19.49
Fisherman	9,539	1.16
Entrepreneur	92,456	11.27

Highest educational attainment		
None	51,703	6.3
Some primary school	139,951	17.05
Completed primary school	252,589	30.78
Completed junior high school	152,758	18.62
Completed senior high school	173,531	21.15
Completed college/university	50,077	6.1

Economic		
Household ever participated in *Raskin*		
No	393,652	47.97
Yes	426,957	52.03

Source is authors' calculation of the IBHR 2013 data.

**Table 3:** Two-part negative binomial logit hurdle model estimates for injuries (*N* = 820,609).

Variables	Two-part negative binomial hurdle			
	Logit		Zero-truncated NB	
	OR	SE	IRR	SE
Spatial				
Region of residence				
Sumatera (Ref.)	1	NA	1	NA
Java-Bali region	1.3934^*∗∗∗*^	0.0155	1.4023^*∗∗∗*^	0.0483
Eastern Indonesia	1.5599^*∗∗∗*^	0.0118	1.1329^*∗∗∗*^	0.0380
Place of residence				
Rural area	1	NA	1	NA
Urban area	1.0290^*∗∗*^	0.0100	1.0437	0.0305
Environmental				
Distance to drinking water source (DWS)				
DWS on premise (Ref.)	1	NA	1	NA
≤100 metres	1.0158	0.0096	1.0304	0.0289
101–1000 metres	1.1388^*∗∗∗*^	0.0188	1.0470	0.0527
>1000 metres	1.0929^*∗∗*^	0.0432	1.1538	0.1338
Household residing in a slum area				
No (Ref.)	1	NA	1	NA
Yes	1.0946^*∗∗∗*^	0.0123	1.0622^*∗*^	0.0356
Sociodemographic				
Sex				
Female (Ref.)	1	NA	1	NA
Male	1.6515^*∗∗∗*^	0.0153	1.1344^*∗∗∗*^	0.0319
Age (in years)				
10–19 (Ref.)	1	NA	1	NA
20–29	0.7549^*∗∗∗*^	0.0134	0.9219^*∗*^	0.0452
30–39	0.6255^*∗∗∗*^	0.0134	0.7913^*∗∗∗*^	0.0517
40–49	0.6053^*∗∗∗*^	0.0137	0.8613^*∗∗*^	0.0591
50–59	0.5979^*∗∗∗*^	0.0143	0.7579^*∗∗∗*^	0.0546
≥60	0.5800^*∗∗∗*^	0.0146	0.7940^*∗∗*^	0.0605
Marital status				
Currently married/living together (Ref.)	1	NA	1	NA
Never married and age ≥ 15 years	1.3300^*∗∗∗*^	0.0208	1.1202^*∗∗*^	0.0563
Never married and age < 15 years	0.9595^*∗*^	0.0216	1.1653^*∗∗*^	0.0770
Bereaved	1.2998^*∗∗∗*^	0.0291	1.1082	0.0779
Divorced/separated	1.2522^*∗∗∗*^	0.0438	1.2696^*∗∗*^	0.1270
Occupation				
Unemployed (Ref.)	1	NA	1	NA
Student	1.0974^*∗∗∗*^	0.0164	0.8865^*∗∗*^	0.0352
Employed	1.1436^*∗∗∗*^	0.0188	0.8314^*∗∗∗*^	0.0361
Farmer	1.0577^*∗∗∗*^	0.0153	1.0546	0.0625
Fisherman	0.9076^*∗∗*^	0.0363	0.9572	0.1270
Entrepreneur	1.0447^*∗∗*^	0.0172	0.8989^*∗*^	0.0497
Highest educational attainment				
None	1.3638^*∗∗∗*^	0.0388	1.8745^*∗∗∗*^	0.1757
Some primary school	1.2439^*∗∗∗*^	0.0307	1.5245^*∗∗∗*^	0.1289
Completed primary school	1.2528^*∗∗∗*^	0.0287	1.3337^*∗∗∗*^	0.1081
Completed junior high school	1.2744^*∗∗∗*^	0.0293	1.1540^*∗*^	0.0942
Completed senior high school	1.1980^*∗∗∗*^	0.0263	1.1592^*∗*^	0.0928
Completed college/university (Ref.)	1	NA	1	NA
Economic				
Household ever participated in *Raskin*				
No (Ref.)	1	NA	1	NA
Yes	1.1207^*∗∗∗*^	0.0107	0.9994	0.0285
Wealth index score (in units)	1.0058	0.0040	0.9612^*∗∗*^	0.0117

*Notes*. NB, negative binomial; OR, odds ratio; IRR, incidence rate ratio; SE, robust standard errors; DWS, drinking water source; NA, not applicable. The symbols *∗∗∗*, *∗∗*, *∗* denote 1, 5, and 10% level of significance, respectively.

Source is authors' calculation of the IBHR 2013 data.
